# Metabolomics in serum of patients with non-advanced age-related macular degeneration reveals aberrations in the glutamine pathway

**DOI:** 10.1371/journal.pone.0218457

**Published:** 2019-06-20

**Authors:** Eveline Kersten, Sascha Dammeier, Soufiane Ajana, Joannes M. M. Groenewoud, Marius Codrea, Franziska Klose, Yara T. Lechanteur, Sascha Fauser, Marius Ueffing, Cécile Delcourt, Carel B. Hoyng, Eiko K. de Jong, Anneke I. den Hollander, EYE-RISK Consortium

**Affiliations:** 1 Department of Ophthalmology, Donders Institute for Brain, Cognition and Behaviour, Radboud university medical center, Nijmegen, the Netherlands; 2 Institute for Ophthalmic Research, Core Facility for Medical Bioanalytics, University of Tübingen, Tübingen, Germany; 3 University of Bordeaux, Inserm, Bordeaux Population Health Research Center, LEHA team, UMR 1219, Bordeaux, France; 4 Department of Epidemiology, Biostatistics, and Health Technology Assessment, Radboud university medical center, Nijmegen, the Netherlands; 5 Quantitative Biology Center, University of Tübingen, Tübingen, Germany; 6 F. Hoffmann - La Roche AG, Basel, Switzerland; 7 Department of Ophthalmology, University Hospital of Cologne, Cologne, Germany; 8 Department of Human Genetics, Donders Institute for Brain, Cognition and Behaviour, Radboud university medical center, Nijmegen, the Netherlands; University of Florida, UNITED STATES

## Abstract

Age-related macular degeneration (AMD) is a common, progressive multifactorial vision-threatening disease and many genetic and environmental risk factors have been identified. The risk of AMD is influenced by lifestyle and diet, which may be reflected by an altered metabolic profile. Therefore, measurements of metabolites could identify biomarkers for AMD, and could aid in identifying high-risk individuals. Hypothesis-free technologies such as metabolomics have a great potential to uncover biomarkers or pathways that contribute to disease pathophysiology. To date, only a limited number of metabolomic studies have been performed in AMD. Here, we aim to contribute to the discovery of novel biomarkers and metabolic pathways for AMD using a targeted metabolomics approach of 188 metabolites. This study focuses on non-advanced AMD, since there is a need for biomarkers for the early stages of disease before severe visual loss has occurred. Targeted metabolomics was performed in 72 patients with early or intermediate AMD and 72 control individuals, and metabolites predictive for AMD were identified by a sparse partial least squares discriminant analysis. In our cohort, we identified four metabolite variables that were most predictive for early and intermediate stages of AMD. Increased glutamine and phosphatidylcholine diacyl C28:1 levels were detected in non-advanced AMD cases compared to controls, while the rate of glutaminolysis and the glutamine to glutamate ratio were reduced in non-advanced AMD. The association of glutamine with non-advanced AMD corroborates a recent report demonstrating an elevated glutamine level in early AMD using a different metabolomics technique. In conclusion, this study indicates that metabolomics is a suitable method for the discovery of biomarker candidates for AMD. In the future, larger metabolomics studies could add to the discovery of novel biomarkers in yet unknown AMD pathways and expand our insights in AMD pathophysiology.

## Introduction

Age-related macular degeneration (AMD) is a common vision-threatening disease affecting the elderly.[1–3] Visual loss in AMD occurs as a result of progressive degenerative events at the centre of the retina, known as the macula. Early AMD is characterized by the accumulation of waste products (drusen) in the macula. Usually, patients experience no or only mild complaints at this stage. As AMD progresses, visual loss occurs and two advanced subtypes of AMD are distinguished: geographic atrophy and choroidal neovascularization, also referred to as wet AMD. Targeting vascular endothelial growth factor, which is central to the disease process of wet AMD, has proven to be a highly effective treatment.[4] However, for the early, intermediate and atrophic stages of AMD, constituting over 80% of AMD patients, no effective treatment exists.

Many environmental and genetic risk factors for AMD have been discovered, including age, smoking, dietary factors (plasma lipids and anti-oxidant levels) and both common and rare genetic variants.[3, 5–9] However, not all individuals with a high genetic risk develop AMD, while some low-risk individuals do develop AMD. Potentially, the disease risk in these AMD patients could be influenced by lifestyle and diet, which may be reflected by their metabolic profile. It has been described that metabolite levels can be influenced by many factors, including age, body-mass index (BMI) and nutrition,[10] factors that are also associated with AMD. Therefore, measurements of metabolites could identify biomarkers for AMD, which could aid in identifying high-risk individuals.

Metabolomics is an hypothesis-free approach that enables simultaneous analysis of large numbers of metabolites, and has the potential to uncover physiological pathways that differ between patients and controls.[11] To date, only a limited number of metabolomic studies have been performed in AMD.[12–17] Two small case-control studies involving a total of 45 and 40 individuals, respectively, showed that individual metabolites and metabolic pathways relevant for AMD pathogenesis can be identified using metabolomics.[13, 15] Another larger study in 396 individuals concluded that, although metabolite changes related to AMD are of low magnitude, they seem to be specific to AMD and further studies are warranted.[12] More recently, a study in 120 individuals indicated that the most significant metabolites belong to the glycerophospholipid pathway.[16]

Here, we aim to contribute to the discovery of novel biomarkers for AMD and uncover clinically relevant metabolic pathways using a targeted metabolomics approach. Ideally, future AMD treatment should be initiated in early stages of the disease to prevent progression to advanced AMD with accompanying visual loss. To identify biomarkers for early stages of the disease, this study focuses on non-advanced AMD.

## Materials and methods

### Study design and study population

Individuals were selected from the European Genetic Database (EUGENDA), a large multicenter database for clinical and molecular analysis of AMD. Disease status was determined based on classification of color fundus photographs, and if available spectral domain optical coherence tomograms and fluorescein angiography by certified graders according to the Cologne Image Reading Center and Laboratory (CIRCL) classification protocol as described previously.[18] In this study, we included cases with non-advanced AMD defined as presence of at least 10 small drusen (<63μm) and pigmentary changes in at least one eye, and absence of central geographic atrophy or choroidal neovascularization in both eyes. Individuals having only pigmentary changes, less than 10 small drusen or without macular abnormalities were classified as control individuals.

Individuals were matched for age, sex, smoking status, body mass index (BMI), number or risk alleles of two prominent common genetic variants in *CFH* (p.Y402H; rs1061170) and *ARMS2* (p.A69S; rs10490924), and complement activation levels (measured as C3d/C3 ratio[19]) to minimize potential confounding effects. Interviewer-assisted questionnaires provided information on lifestyle, dietary habits and other environmental factors. For metabolomic analyses in this study, 72 AMD cases and 72 controls were selected (total *n* = 144).

This study was approved by the local ethical committees at both sites of patient recruitment, the Radboud university medical center and the University Hospital of Cologne, and was performed in accordance with the tenets of the Declaration of Helsinki. Written informed consent was provided by all individuals.

### Serum collection and genotyping

Venous blood samples were collected from all individuals in a non-fasting state at time of enrolment in EUGENDA. Serum was obtained using standardized coagulation and centrifugation procedures, and subsequently stored at -80°C within one hour after collection until analysis.

Genomic DNA was extracted from peripheral blood leukocytes, and genotyping of single nucleotide polymorphisms (SNPs) in the *CFH* (rs1061170) and *ARMS2* (rs10490924) genes was performed using competitive allele-specific PCR assays (KASPar SNP Genotyping System, KBiosciences).

### Targeted metabolomics

Targeted identification and quantification of 188 metabolites (S1 Table) was achieved by executing the mass spectrometric acquisition methods as provided by the AbsoluteIDQ p180 kit (Biocrates Life Sciences, Innsbruck, Austria) with some modifications. In brief, after thawing serum samples were centrifuged at 2750x*g* at 4°C for 5 minutes. Subsequently, 10 μL of each sample, quality controls, calibrators and zero samples were pipeted on the upper filter inlays of the AbsoluteIDQ multiwell plate, which already contained the proprietary mixture of internal standards. After drying, each filter inlay was incubated with 50 μL of 5% (v/v) phenylisothiocyanate in a solvent solution consisting of ethanol, water and pyridine (1:1:1) at room temperature for 20 minutes. The final extraction of the metabolites was performed for each well with 300 μL of methanol containing 5 mM ammonium acetate using a positive pressure device (Waters, Eschborn, Germany). Instead of using a conventional HPLC-MS system the analyses were performed on an Eksigent 200 microLC chromatography system (ABSciex, Darmstadt, Germany) coupled to a 6500 QTRAP (ABSciex, Darmstadt, Germany). To detect amino acids and biogenic amines, 50 μL of the metabolite extract were diluted in 350 μL of 50% methanol. Chromatography was performed using two running solvents (A: water, 0.2% formic acid; B: acetonitrile, 0.2% formic acid). Two μL of the diluted metabolite extracts were resolved on an Acquity UPLC BEH C18, 1.0 x 50 mm (120 A) reverse phase column (Waters, Eschborn, Germany) using a linear gradient from 2% B to 40% B in 3.5 minutes, from 40% B to 80% B in 1.5 minutes, and to 100% B in 0.1 min at 30 μL/min.

To determine the content of glycerophospholipids, hexoses and acylcarnitines, 50 μL of the metabolite extract were diluted with 450 μL methanol. Five μL of this dilution were analyzed in the mass spectrometer by direct infusion using the acquisition parameters as given by the manufacturer’s manual. Two injections were done to acquire data in positive and negative mode separately.

### Quality control

Technical quality control steps were undertaken before statistical analyses. Individual analytical batches were normalized to at least 3 replicates of the identical plasma quality control provided by the kit manufacturer to account for plate-to-plate variability. All metabolites that exhibited concentration values below limit of detection (as defined by the analytical specifications) in more than 50% of the measurements were omitted from the dataset (S1 Table).

### Statistical analyses

To compare demographic characteristics of the two groups, one-way ANOVA and chi-squared tests were performed. After quality control 153 metabolites remained available for statistical analyses (S1 Table). Additionally, various derivative variables were created based on the metabolite levels (n = 57; S2 Table). To evaluate potential different mean levels of the metabolites between cases and controls non-parametric wilcoxon signed-rank tests designed for matched samples in case of non-normally distributed data were performed for each variable (S3 Table). Due to the large number of variables to be evaluated, we used a sparse partial least squares discriminant analysis (sPLSda) to perform variable selection while taking into account the correlations between the variables. This approach aims at combining variable selection and dimension reduction in a one-step procedure.[20] We considered one latent dimension since we are predicting a univariate binary outcome and to facilitate interpretation of the model.[20] The optimal tuning parameters (i.e., number of selected predictors) were estimated using a leave one out cross-validation strategy. Thereafter, a logistic regression was performed on the selected predictors resulting from the sPLSda to reduce the bias induced by shrinkage.[21] We performed these analyses on the entire dataset (including the created variables) and on the crude metabolites only. The discriminative accuracy of each model (resulting from the entire dataset and from the crude metabolites, respectively) was evaluated using receiver operating characteristic (ROC) curves and calculation of their corresponding area under the curve (AUC). Statistical tests were performed using R statistical software (R Core Team (2016). R: A language and environment for statistical computing. R Foundation for Statistical Computing, Vienna, Austria. URL https://www.R-project.org/). We used the cran R package “mixOmics” to train and to test the sPLSda.

## Results

Serum samples of 72 cases and 72 control individuals were selected for metabolomic analyses. Demographic characteristics and other potential confounding factors in our cohort are provided in Table 1. No significant differences between the groups were detected for sex, age, smoking status, BMI, diabetic status, diet, complement activation levels and number of *CFH* rs1061170 and *ARMS2* rs10490924 risk alleles.

**Table 1:** Patient characteristics of AMD cases and control individuals.

		AMD cases (n = 72)	Control individuals (n = 72)	*P*-value
**Sex**	Male	26 (36.1%)	28 (38.9%)	
	Female	46 (63.9%)	44 (61.1%)	0.73
**Age (mean years ±SD)**		72.65±7.30	70.64±5.27	0.06
**Smoking status**	Never	34 (47.2%)	37 (51.4%)	
	Past	38 (52.8%)	35 (48.6%)	
	Current	0 (0%)	0 (0%)	0.62
**BMI (kg/m**^**2**^**)**	<20	3 (4.2%)	3 (4.2%)	
	20–25	35 (48.6%)	29 (40.3%)	
	25–30	28 (38.9%)	33 (45.8%)	
	>30	6 (8.3%)	7 (9.7%)	0.79
Diabetes Mellitus*	Absent	64 (90.1%)	64 (91.4%)	
	Present	7 (9.9%)	6 (8.6%)	0.79
**Diet**	Regular diet	65 (94.2%)	66 (95.7%)	
	Vegetarian diet**	4 (5.8%)	3 (4.3%)	0.70
Complement activation (mean±SD***)	C3d/C3 ratio	1.54±0.40	1.47±0.44	0.37
***CFH*, rs1061170 (C)**	MAF	44.4	45.1	0.93
***ARMS2*, rs10490924 (T)**	MAF	34.7	32.6	0.74

*Self-reported diagnosis

**Vegetarian diet when participant indicated to (almost) never eat fish and red meat

***For the purpose of analyses data was transformed to the natural logarithm

Abbreviations: SD, standard deviation; BMI, body mass index; MAF, minor allele frequency.

**Fig 1:**
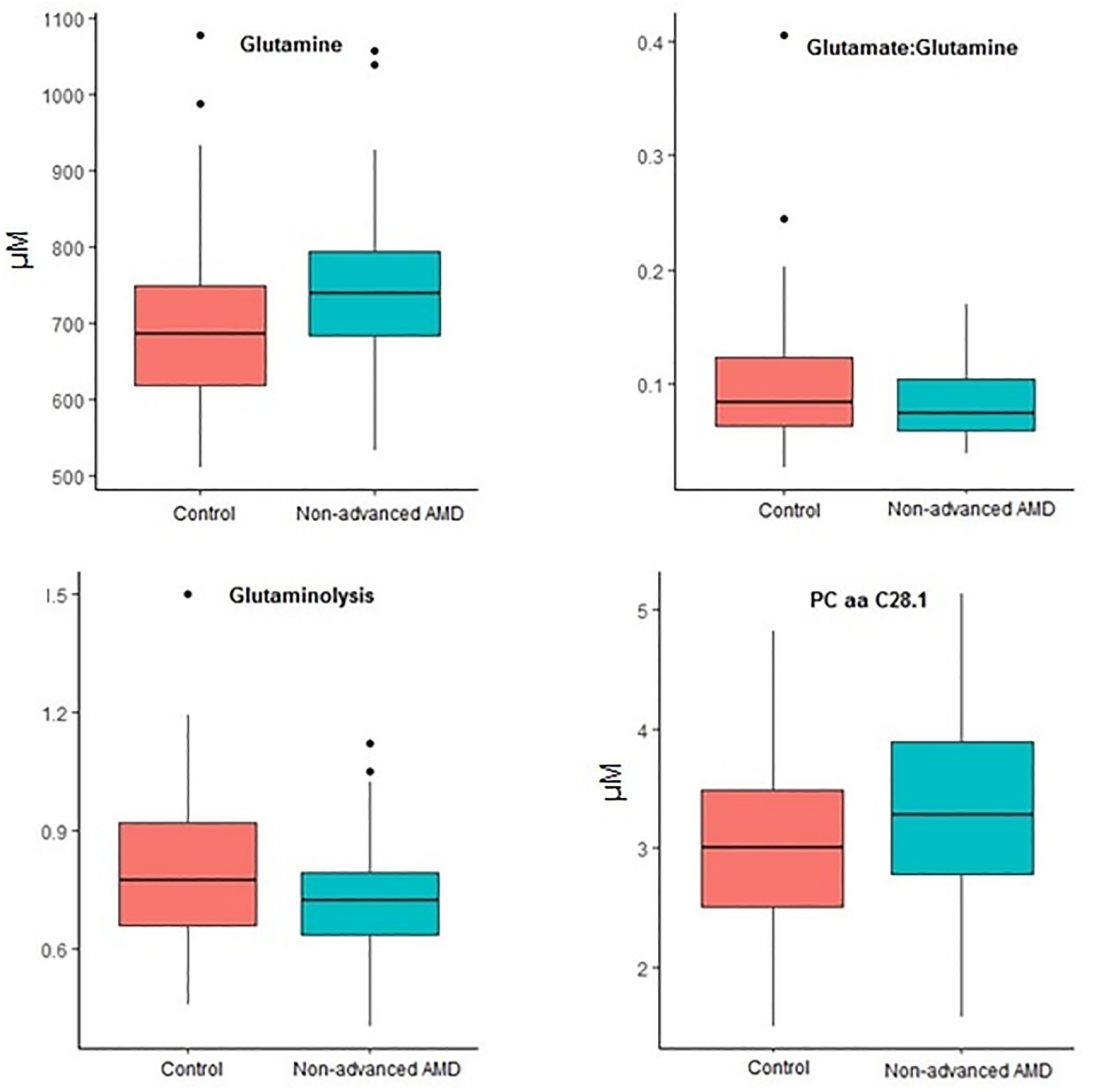
Boxplots of the four metabolite predictors for non-advanced AMD from sPLSda. All metabolites were measured in μM. Glutaminolysis is measured as (c_Ala_+c_Asp_+c_Glu_)/c_Gln_.

**Fig 2:**
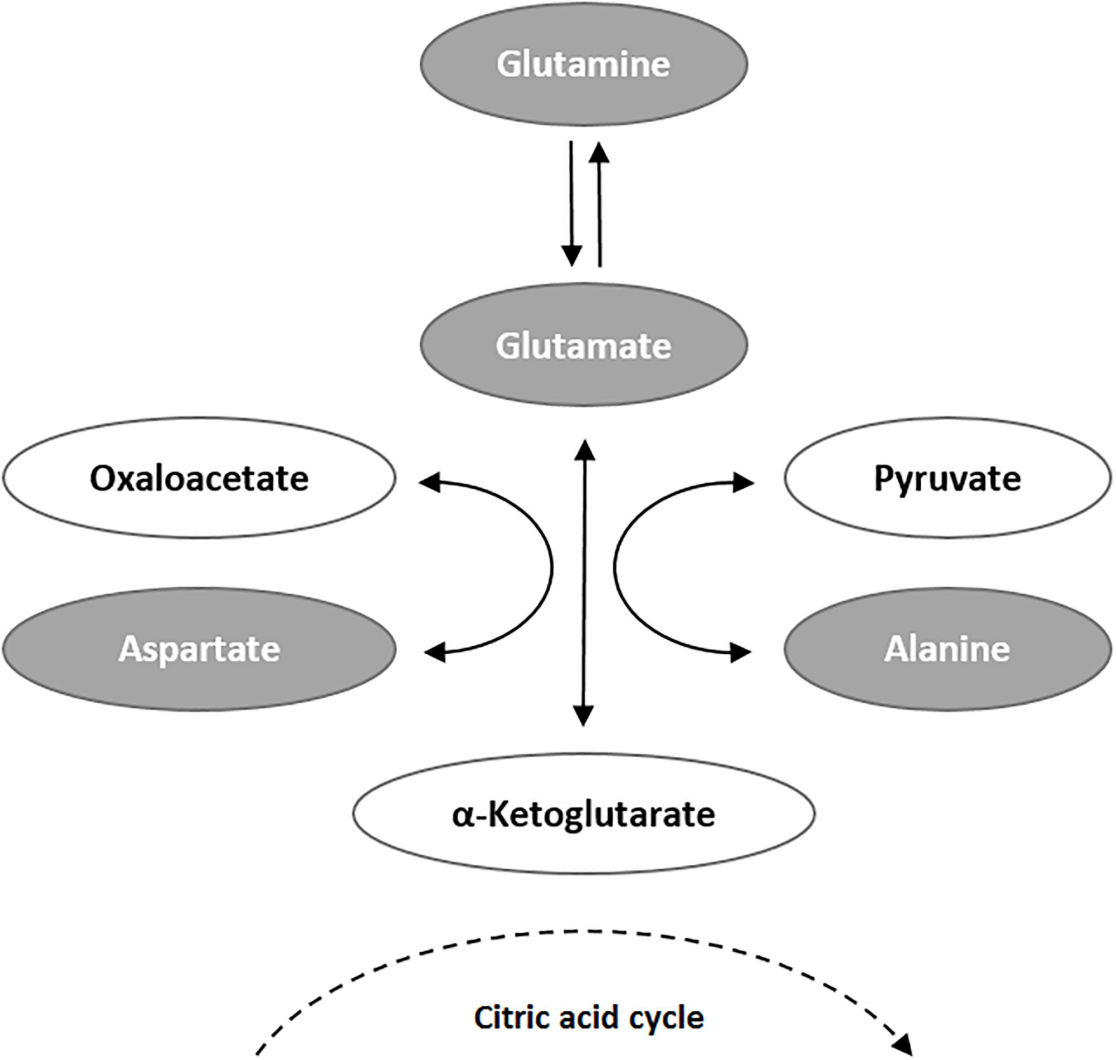
Metabolic conversion of glutamine. Glutaminolysis, the metabolic conversion of glutamine to glutamate, aspartate and alanine, represents an alternative pathway to supply the mitochondrial citric acid cycle with a surplus of α-ketoglutarate. As this pathway is preferentially used by proliferating tissue, glutaminolysis measured as (c_Ala_+c_Asp_+c_Glu_)/c_Gln_ is increased in tumor tissue.[22] Metabolites determined in this study are marked in grey.

Next, we performed sPLSda to select the most predictive variables from our dataset, including all 153 measured metabolites and 57 derivative variables. This approach selected four relevant predictors for non-advanced AMD: glutamine, glutamate:glutamine ratio, glutaminolysis, and phosphatidylcholine diacyl C28:1 (PC aa C28.1) (Table 2; Fig 1). Two of these predictors are derivative variables that were created from measured metabolite levels, which both involved glutamine: the rate of glutaminolysis was expressed by the ratio of the sum of the potential glutamine conversion products (aspartate, alanine, glutamate) to glutamine (Fig 2), and another measure of glutamine metabolism was defined as the ratio between glutamine and glutamate (Glu:Gln ratio).

**Table 2:** Metabolite predictors for non-advanced AMD from sPLSda.

	Estimate (β)*	Odds ratio**
Glutamine (μM)	0.0037	1.004
Glu:Gln ratio	-2.79	0.061
Glutaminolysis	-1.73	0.177
PC.aa.C28.1 (μM)	0.62	1.858

*The estimate associated to a predictor represents the change in the log odds per unit change in this predictor if all other predictors are held constant.

** The odds ratio represents the odds to be a case given a particular exposure, compared to the odds of being a case in the absence of that exposure.

The distributions of these variables in non-advanced AMD cases and control individuals are illustrated in Fig 1. A higher mean glutamine level was detected in non-advanced AMD cases (746.33 μM) compared to controls (695.0 μM). The mean rate of glutaminolysis and the Glu:Gln ratio were reduced in non-advanced AMD cases (0.73 and 0.08, respectively) compared to controls (0.80 and 0.10, respectively). Besides glutamine levels, mean levels of the metabolites used to create these custom metabolic indicators were not significantly different between cases and controls, although glutamate levels were slightly higher in controls (S3 Table). The mean level of phosphatidylcholine diacyl C28:1 was elevated in non-advanced AMD cases (3.35 μM) compared to controls (3.04 μM).

When performing sPLSda on the measured metabolites only (excluding the derivative variables), glutamine levels were the most predictive for non-advanced AMD (OR 1.005).

**Fig 3:**
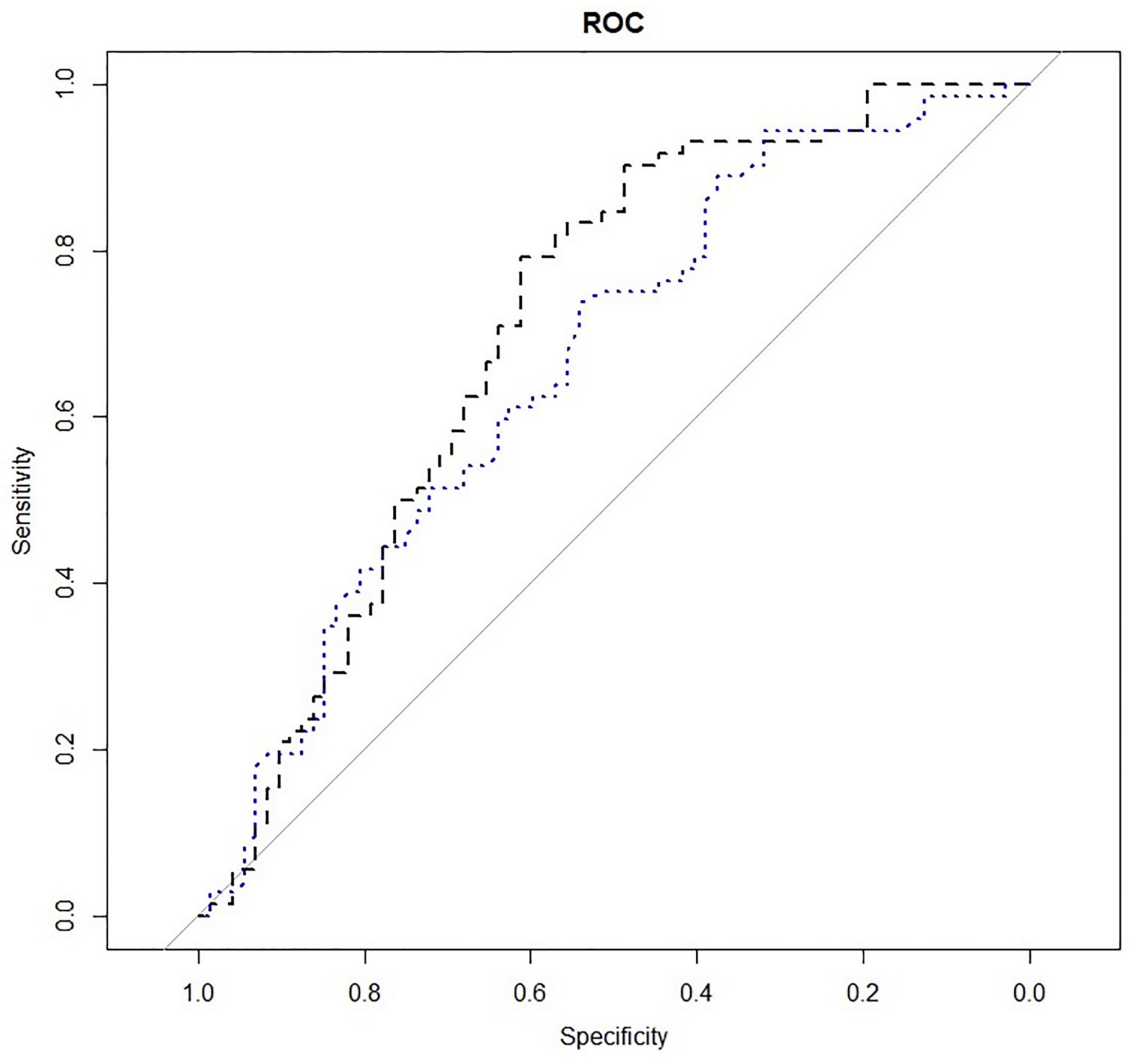
Receiver operating characteristic curves of the logistic regression models obtained from the entire dataset including derivative variables (black curve) and from crude dataset (blue curve).

The ability to discriminate between cases and controls based on the predictors resulting from sPLSda of both the entire dataset (including derivative variables) and crude dataset (excluding derivative variables), respectively, was evaluated using ROC curves and calculation of the corresponding area under the curves (Fig 3). The model resulting from sPLSda on the entire dataset (including glutamine, Glu:Gln ratio, glutaminolysis, and PC.aa.C28.1) showed superior performance (AUC of 0.71, 95% CI: 0.62–0.79) compared to the model including glutamine only as predictor (AUC of 0.66, 95% CI: 0.57–0.75).

## Discussion

In the present study, we investigated potential differences of the metabolome between non-advanced AMD patients and control individuals using a targeted metabolomics approach. Four variables were identified by sPLSda as the most predictive features to discriminate between non-advanced AMD patients and control individuals, including glutamine, glutamine-related variables and a glycerophospholipid (PC aa C28.1). These results are in line with previous studies describing metabolic differences between AMD patients and controls.[12, 13, 15] Of particular interest is the slightly increased glutamine level in AMD patients, which independently corroborates a recent report demonstrating an elevated glutamine level in early AMD using a different metabolomics technique.[12] Also, other measures of glutamine metabolism in our study were indicative of a possible association between glutamine and AMD. In AMD patients, both glutaminolysis (the ratio of glutamate, aspartate and alanine to glutamine) as well as the Glu:Gln ratio were decreased, although these effects were driven mainly by elevated levels of glutamine. Glutamine is a nonessential amino acid necessary to sustain immune competence,[23] and immunological processes are at the heart of AMD pathology.[24, 25] It remains to be investigated whether increased glutamine in serum is a physiological response to a higher demand from the immune system or a result of increased protein catabolism, decreased clearance of glutamine, increased dietary intake of glutamine, or other mechanisms. Additionally, one might argue that the change in plasma glutamine levels might not reflect glutamine changes in RPE or retina, as the glutamine/glutamate system in the central nervous system (CNS) is characterized by its local, partly autonomous regulation. There is evidence that glutamate could not readily cross the blood-brain barrier[26], this implicates that the glutamate concentration in the brain is independent from changes of plasma glutamate. However, there is also evidence that the glutamate/glutamine ratio in systemic body fluids could be explicitly affected by alterations of the CNS metabolism, e.g. in neuropathological disorders. For instance, it has been reported repeatedly that autism induces significant changes in the Glu/Gln ratio in serum[27]. Hence blood glutamate scavenging has been proposed as a treatment option to support neuroprotection in general[28]. With regard to the above, and the metabolic changes of glutamine reported by another metabolomic study[12] we are strongly encouraged to propose a significant influence of the retinal tissue on systemic metabolism.

The observed association between a glycerophospholipid (PC aa C28.1) and non-advanced AMD is interesting because alterations in concentrations of these species have been implicated in a variety of metabolic diseases and pathomechanisms.[29–31] Glycerophospholipids and sphingomyelin are constituents of cell membranes and myelin sheaths, and phosphatidylcholines are the main constituents of lipoproteins,[32] which have been described to be relevant for AMD pathology.[33, 34] Although literature is not entirely consistent, multiple studies have associated higher high-density lipoprotein cholesterol levels with increased risk of AMD.[8, 25, 34–37] Furthermore, it is conceivable that not only levels of lipoprotein classes, but also their molecular composition in terms of glycerophospholipid species could be potential biomarkers for AMD development, as has been reported for other conditions, such as arterial hypertension.[38] Therefore in-depth lipidomics studies that cover a wider range of lipoprotein subclasses and their molecular constituents are required to corroborate our findings and to further explore the relationship between lipids, lipoprotein dynamics and AMD pathology.

It must be noted that the effect sizes of the identified AMD-associated metabolites are small, consistent with slightly altered metabolic profiles of AMD patients compared to controls. Due to the limited sample size of the current study, we might have been unable to detect smaller associations and therefore larger studies are warranted. A strength of our study is that the study groups were carefully selected and matched on potential confounders including age, sex, smoking status and BMI. Due to this strict patient stratification, differences in metabolites likely reflect true differences in metabolic profiles between AMD cases and controls. Additionally, our study included patients with non-advanced AMD only, which allows for the identification of potentially relevant biomarkers already in an early stage of the disease.

Of note, the samples used for this study were collected at time of enrolment in EUGENDA and were not specifically collected for the current study. Because of possible influences of diet on the metabolome, the use of non-fasting samples in this study might not be ideal. However, although the reproducibility of metabolite levels over time was previously reported to be lower using non-fasting samples compared to fasting samples, in general the reliability of metabolites was not significantly different when comparing fasting versus non-fasting samples.[39]

In summary, the findings of this study indicate that metabolomics is a suitable method for the discovery of biomarkers in AMD. Using a targeted approach, several metabolites were identified as candidate biomarkers for AMD with glutamine being the most promising, which may serve as potential targets for future interventions. Larger metabolomic studies are needed to further elucidate the metabolic profile of AMD patients. Additionally, untargeted metabolomic studies could provide novel biomarkers in yet unknown AMD pathways and expand our insights in AMD pathophysiology.

## Supporting information

S1 Table List of metabolites. (DOCX)

S2 Table List of custom metabolic indicator variables. (DOCX)

S3 Table Comparison of mean levels of all variables between cases and controls. (DOCX)

S1 Dataset Anonymized dataset. (XLSX)
