# Supplementary material for: Metabolomics in serum of patients with non-advanced age-related macular degeneration reveals aberrations in the glutamine pathway
Source: PLoS One. 2019 Jun 20;14(6):e0218457. doi: 10.1371/journal.pone.0218457 (PMC6586309; doi:10.1371/journal.pone.0218457)
Supplement: S1 Table — (DOCX) [file pone.0218457.s001.docx]

**S1 Table. List of metabolites**

| **Metabolite name** | **Abbreviation** | **Passed QC?** |
| --- | --- | --- |
| **Aminoacids (n=21)** | | |
| Alanine | Ala | Yes |
| Arginine | Arg | Yes |
| Asparagine | Asn | Yes |
| Aspartate | Asp | Yes |
| Citrulline | Cit | Yes |
| Glutamine | Gln | Yes |
| Glutamate | Glu | Yes |
| Glycine | Gly | Yes |
| Histidine | His | Yes |
| Isoleucine | Ile | Yes |
| Leucine | Leu | Yes |
| Lysine | Lys | Yes |
| Methionine | Met | Yes |
| Ornithine | Orn | Yes |
| Phenylalanine | Phe | Yes |
| Proline | Pro | Yes |
| Serine | Ser | Yes |
| Threonine | Thr | Yes |
| Tryptophan | Trp | Yes |
| Tyrosine | Tyr | Yes |
| Valine | Val | Yes |
| **Biogenic amines (n=21)** | | |
| Acetylornithine | Ac-Orn | < LOD |
| Asymmetric dimethylarginine | ADMA | Yes |
| alpha-Aminoadipic acid | alpha-AAA | < LOD |
| cis-4-Hydroxyproline | c4-OH-Pro | < LOD |
| Carnosine | Carnosine | < LOD |
| Creatinine | Creatinine | Yes |
| DOPA | DOPA | < LOD |
| Dopamine | Dopamine | < LOD |
| Histamine | Histamine | < LOD |
| Kynurenine | Kynurenine | Yes |
| Methioninesulfoxide | Met-SO | < LOD |
| Nitrotyrosine | Nitro-Tyr | < LOD |
| Phenylethylamine | PEA | < LOD |
| Putrescine | Putrescine | Yes |
| Sarcosine | Sarcosine | Yes |
| Serotonin | Serotonin | Yes |
| Spermidine | Spermidine | < LOD |
| Spermine | Spermine | < LOD |
| trans-OH-Pro | t4-OH-Pro | < LOD |
| Taurine | Taurine | Yes |
| Symmetric dimethylarginine | SDMA | Yes |
| **Acylcarnitines (n=40)** | | |
| Carnitine | C0 | Yes |
| Acetylcarnitine | C2 | Yes |
| Propionylcarnitine | C3 | Yes |
| Propenoylcarnitine | C3:1 | < LOD |
| Hydroxypropionylcarnitine | C3-OH | < LOD |
| Butyrylcarnitine | C4 | Yes |
| Butenylcarnitine | C4:1 | Yes |
| Hydroxybutyrylcarnitine | C4-OH (C3-DC) | Yes |
| Valerylcarnitine | C5 | Yes |
| Tiglylcarnitine | C5:1 | Yes |
| Glutaconylcarnitine | C5:1-DC | Yes |
| Glutarylcarnitine (Hydroxyhexanoylcarnitine) | C5-DC (C6-OH) | Yes |
| Methylglutarylcarnitine | C5-M-DC | < LOD |
| Hydroxyvalerylcarnitine  (Methylmalonylcarnitine) | C5-OH (C3-DC-M) | < LOD |
| Hexanoylcarnitine  (Fumarylcarnitine) | C6 (C4:1-DC) | Yes |
| Hexenoylcarnitine | C6:1 | < LOD |
| Pimelylcarnitine | C7-DC | < LOD |
| Octanoylcarnitine | C8 | Yes |
| Nonaylcarnitine | C9 | Yes |
| Decanoylcarnitine | C10 | Yes |
| Decenoylcarnitine | C10:1 | Yes |
| Decadienylcarnitine | C10:2 | < LOD |
| Dodecanoylcarnitine | C12 | Yes |
| Dodecenoylcarnitine | C12:1 | Yes |
| Dodecanedioylcarnitine | C12-DC | < LOD |
| Tetradecanoylcarnitine | C14 | Yes |
| Tetradecenoylcarnitine | C14:1 | Yes |
| Hydroxytetradecenoylcarnitine | C14:1-OH | < LOD |
| Tetradecadienylcarnitine | C14:2 | Yes |
| Hydroxytetradecadienylcarnitine | C14:2-OH | < LOD |
| Hexadecanoylcarnitine | C16 | Yes |
| Hexadecenoylcarnitine | C16:1 | < LOD |
| Hydroxyhexadecenoylcarnitine | C16:1-OH | < LOD |
| Hexadecadienylcarnitine | C16:2 | < LOD |
| Hydroxyhexadecadienylcarnitine | C16:2-OH | < LOD |
| Hydroxyhexadecanoylcarnitine | C16-OH | < LOD |
| Octadecanoylcarnitine | C18 | Yes |
| Octadecenoylcarnitine | C18:1 | Yes |
| Hydroxyoctadecenoylcarnitine | C18:1-OH | < LOD |
| Octadecadienylcarnitine | C18:2 | Yes |
| **Glycerophospholipids (n=90)** | | |
| lysophosphatidylcholine acyl C14:0 | lysoPC a C14:0 | < LOD |
| lysophosphatidylcholine acyl C16:0 | lysoPC a C16:0 | Yes |
| lysophosphatidylcholine acyl C16:1 | lysoPC a C16:1 | Yes |
| lysophosphatidylcholine acyl C17:0 | lysoPC a C17:0 | Yes |
| lysophosphatidylcholine acyl C18:0 | lysoPC a C18:0 | Yes |
| lysophosphatidylcholine acyl C18:1 | lysoPC a C18:1 | Yes |
| lysophosphatidylcholine acyl C18:2 | lysoPC a C18:2 | Yes |
| lysophosphatidylcholine acyl C20:3 | lysoPC a C20:3 | Yes |
| lysophosphatidylcholine acyl C20:4 | lysoPC a C20:4 | Yes |
| lysophosphatidylcholine acyl C24:0 | lysoPC a C24:0 | Yes |
| lysophosphatidylcholine acyl C26:0 | lysoPC a C26:0 | Yes |
| lysophosphatidylcholine acyl C26:1 | lysoPC a C26:1 | Yes |
| lysophosphatidylcholine acyl C28:0 | lysoPC a C28:0 | Yes |
| lysophosphatidylcholine acyl C28:1 | lysoPC a C28:1 | Yes |
| Phosphatidylcholine diacyl C24:0 | PC aa C24:0 | Yes |
| Phosphatidylcholine diacyl C26:0 | PC aa C26:0 | < LOD |
| Phosphatidylcholine diacyl C28:1 | PC aa C28:1 | Yes |
| Phosphatidylcholine diacyl C30:0 | PC aa C30:0 | Yes |
| Phosphatidylcholine diacyl C30:2 | PC aa C30:2 | < LOD |
| Phosphatidylcholine diacyl C32:0 | PC aa C32:0 | Yes |
| Phosphatidylcholine diacyl C32:1 | PC aa C32:1 | Yes |
| Phosphatidylcholine diacyl C32:2 | PC aa C32:2 | Yes |
| Phosphatidylcholine diacyl C32:3 | PC aa C32:3 | Yes |
| Phosphatidylcholine diacyl C34:1 | PC aa C34:1 | Yes |
| Phosphatidylcholine diacyl C34:2 | PC aa C34:2 | Yes |
| Phosphatidylcholine diacyl C34:3 | PC aa C34:3 | Yes |
| Phosphatidylcholine diacyl C34:4 | PC aa C34:4 | Yes |
| Phosphatidylcholine diacyl C36:0 | PC aa C36:0 | Yes |
| Phosphatidylcholine diacyl C36:1 | PC aa C36:1 | Yes |
| Phosphatidylcholine diacyl C36:2 | PC aa C36:2 | Yes |
| Phosphatidylcholine diacyl C36:3 | PC aa C36:3 | Yes |
| Phosphatidylcholine diacyl C36:4 | PC aa C36:4 | Yes |
| Phosphatidylcholine diacyl C36:5 | PC aa C36:5 | Yes |
| Phosphatidylcholine diacyl C36:6 | PC aa C36:6 | Yes |
| Phosphatidylcholine diacyl C38:0 | PC aa C38:0 | Yes |
| Phosphatidylcholine diacyl C38:1 | PC aa C38:1 | Yes |
| Phosphatidylcholine diacyl C38:3 | PC aa C38:3 | Yes |
| Phosphatidylcholine diacyl C38:4 | PC aa C38:4 | Yes |
| Phosphatidylcholine diacyl C38:5 | PC aa C38:5 | Yes |
| Phosphatidylcholine diacyl C38:6 | PC aa C38:6 | Yes |
| Phosphatidylcholine diacyl C40:1 | PC aa C40:1 | < LOD |
| Phosphatidylcholine diacyl C40:2 | PC aa C40:2 | Yes |
| Phosphatidylcholine diacyl C40:3 | PC aa C40:3 | Yes |
| Phosphatidylcholine diacyl C40:4 | PC aa C40:4 | Yes |
| Phosphatidylcholine diacyl C40:5 | PC aa C40:5 | Yes |
| Phosphatidylcholine diacyl C40:6 | PC aa C40:6 | Yes |
| Phosphatidylcholine diacyl C42:0 | PC aa C42:0 | Yes |
| Phosphatidylcholine diacyl C42:1 | PC aa C42:1 | Yes |
| Phosphatidylcholine diacyl C42:2 | PC aa C42:2 | Yes |
| Phosphatidylcholine diacyl C42:4 | PC aa C42:4 | Yes |
| Phosphatidylcholine diacyl C42:5 | PC aa C42:5 | Yes |
| Phosphatidylcholine diacyl C42:6 | PC aa C42:6 | Yes |
| Phosphatidylcholine acyl-alkyl C30:0 | PC ae C30:0 | Yes |
| Phosphatidylcholine acyl-alkyl C30:1 | PC ae C30:1 | Yes |
| Phosphatidylcholine acyl-alkyl C30:2 | PC ae C30:2 | Yes |
| Phosphatidylcholine acyl-alkyl C32:1 | PC ae C32:1 | Yes |
| Phosphatidylcholine acyl-alkyl C32:2 | PC ae C32:2 | Yes |
| Phosphatidylcholine acyl-alkyl C34:0 | PC ae C34:0 | Yes |
| Phosphatidylcholine acyl-alkyl C34:1 | PC ae C34:1 | Yes |
| Phosphatidylcholine acyl-alkyl C34:2 | PC ae C34:2 | Yes |
| Phosphatidylcholine acyl-alkyl C34:3 | PC ae C34:3 | Yes |
| Phosphatidylcholine acyl-alkyl C36:0 | PC ae C36:0 | Yes |
| Phosphatidylcholine acyl-alkyl C36:1 | PC ae C36:1 | Yes |
| Phosphatidylcholine acyl-alkyl C36:2 | PC ae C36:2 | Yes |
| Phosphatidylcholine acyl-alkyl C36:3 | PC ae C36:3 | Yes |
| Phosphatidylcholine acyl-alkyl C36:4 | PC ae C36:4 | Yes |
| Phosphatidylcholine acyl-alkyl C36:5 | PC ae C36:5 | Yes |
| Phosphatidylcholine acyl-alkyl C38:0 | PC ae C38:0 | Yes |
| Phosphatidylcholine acyl-alkyl C38:1 | PC ae C38:1 | Yes |
| Phosphatidylcholine acyl-alkyl C38:2 | PC ae C38:2 | Yes |
| Phosphatidylcholine acyl-alkyl C38:3 | PC ae C38:3 | Yes |
| Phosphatidylcholine acyl-alkyl C38:4 | PC ae C38:4 | Yes |
| Phosphatidylcholine acyl-alkyl C38:5 | PC ae C38:5 | Yes |
| Phosphatidylcholine acyl-alkyl C38:6 | PC ae C38:6 | Yes |
| Phosphatidylcholine acyl-alkyl C40:1 | PC ae C40:1 | Yes |
| Phosphatidylcholine acyl-alkyl C40:2 | PC ae C40:2 | Yes |
| Phosphatidylcholine acyl-alkyl C40:3 | PC ae C40:3 | Yes |
| Phosphatidylcholine acyl-alkyl C40:4 | PC ae C40:4 | Yes |
| Phosphatidylcholine acyl-alkyl C40:5 | PC ae C40:5 | Yes |
| Phosphatidylcholine acyl-alkyl C40:6 | PC ae C40:6 | Yes |
| Phosphatidylcholine acyl-alkyl C42:0 | PC ae C42:0 | < LOD |
| Phosphatidylcholine acyl-alkyl C42:1 | PC ae C42:1 | Yes |
| Phosphatidylcholine acyl-alkyl C42:2 | PC ae C42:2 | Yes |
| Phosphatidylcholine acyl-alkyl C42:3 | PC ae C42:3 | Yes |
| Phosphatidylcholine acyl-alkyl C42:4 | PC ae C42:4 | Yes |
| Phosphatidylcholine acyl-alkyl C42:5 | PC ae C42:5 | Yes |
| Phosphatidylcholine acyl-alkyl C44:3 | PC ae C44:3 | Yes |
| Phosphatidylcholine acyl-alkyl C44:4 | PC ae C44:4 | Yes |
| Phosphatidylcholine acyl-alkyl C44:5 | PC ae C44:5 | Yes |
| Phosphatidylcholine acyl-alkyl C44:6 | PC ae C44:6 | Yes |
| **Sphingolipids (n=15)** | | |
| Hydroxysphingomyelin C14:1 | SM (OH) C14:1 | Yes |
| Hydroxysphingomyelin C16:1 | SM (OH) C16:1 | Yes |
| Hydroxysphingomyelin C22:1 | SM (OH) C22:1 | Yes |
| Hydroxysphingomyelin C22:2 | SM (OH) C22:2 | Yes |
| Hydroxysphingomyelin C24:1 | SM (OH) C24:1 | Yes |
| Sphingomyelin C16:0 | SM C16:0 | Yes |
| Sphingomyelin C16:1 | SM C16:1 | Yes |
| Sphingomyelin C18:0 | SM C18:0 | Yes |
| Sphingomyelin C18:1 | SM C18:1 | Yes |
| Sphingomyelin C20:2 | SM C20:2 | Yes |
| Sphingomyelin C22:3 | SM C22:3 | < LOD |
| Sphingomyelin C24:0 | SM C24:0 | Yes |
| Sphingomyelin C24:1 | SM C24:1 | Yes |
| Sphingomyelin C26:0 | SM C26:0 | Yes |
| Sphingomyelin C26:1 | SM C26:1 | Yes |
| **Monosaccharides (n=1)** | | |
| Sum of hexoses | H1 | Yes |

Abbreviations: LOD = limit of detection.

*Notation of fatty acid chains take the form “C x:y,” where “x” is the number of carbon atoms and “y” the number of double bonds in the fatty acid.
