# Supplementary material for: Metabolomics in serum of patients with non-advanced age-related macular degeneration reveals aberrations in the glutamine pathway
Source: PLoS One. 2019 Jun 20;14(6):e0218457. doi: 10.1371/journal.pone.0218457 (PMC6586309; doi:10.1371/journal.pone.0218457)
Supplement: S2 Table — (DOCX) [file pone.0218457.s002.docx]

**S2 Table. List of custom metabolic indicator variables**

| **Custom metabolic indicator** | **Calculation** |
| --- | --- |
| AAA | Sum of aromatic amino acids (Phe, Tyr, Trp) |
| ADMA/Arg | Ratio of Asymmetric dimethylarginine to Arginine |
| Arg/(Arg+Orn) | Ratio of Arginine to (Arginine+Ornithine) |
| BCAA | Sum of branched chain amino acids (Val, Leu, Ile) |
| Cit/Arg | Ratio of Citrulline to Arginine |
| Cit/Orn | Ratio of Citrulline to Ornithine |
| Essential AA | Total of essential aminoacids |
| Fisher ratio | Ratio of BCAA to AAA |
| Glu/Gln | Ratio of Glutamate to Glutamine |
| Glucogenic AA | Sum of selected amino acids (Ala, Gly, Ser) |
| Glutaminolysis | Ratio of (Asparagine+Alanine+Glutamate) to Glutamine |
| Gly/Arg | Ratio of Glycine to Arginine |
| Gly/Gln | Ratio of Glycine to Glutamine |
| Gly/His | Ratio of Glycine to Histidine |
| Gly/Ser | Ratio of Glycine to Serine |
| Glycolysis | Sum of selected amino acids (Ala, Gly, Ser) |
| Kynurenine/Trp | Ratio of Kynurenine to Tryptophan |
| Non essential AA | Total of non essential aminoacids |
| Orn/Arg | Ratio of Ornithine to Arginine |
| Orn/Ser | Ratio of Ornithine to Serine |
| Putrescine/Orn | Ratio of Putrescine to Ornithine |
| SDMA/Arg | Ratio of Symmetric dimethylarginine to Arginine |
| Serotonin/Trp | Ratio of Serotonin to Tryptophan |
| Thr/Ser | Ratio of Threonine to Serine |
| Total AA | Total of aminoacids |
| Total DMA/Arg | Ratio of (SDMA+ADMA) to Arginine |
| Tyr/Phe | Ratio of Tyrosine to Phenylalanine |
| (C2+C3)/C0 | Ratio of (Acetylcarnitine+Propionylcarnitine) to Carnitine |
| C18/C18:1 | Ratio of Octadecanoylcarnitine to Octadecenoylcarnitine |
| C2/C0 | Ratio of Acetylcarnitine to Carnitine |
| C3/C4 | Ratio of Propionylcarnitine to Butyrylcarnitine |
| C4/C0 | Ratio of Butyrylcarnitine to Carnitine |
| C4/C5 | Ratio of Butyrylcarnitine to Valerylcarnitine |
| CPT-I ratio | Ratio of (Hexadecanoylcarnitine+Octadecanoylcarnitine) to Carnitine |
| lysoPC a C16:0/lysoPC a C16:1 | Ratio of lysophosphatidylcholine acyl C16:0 to lysophosphatidylcholine acyl C16:1 |
| lysoPC a C20:4/lysoPC a C20:3 | Ratio of lysophosphatidylcholine acyl C20:4 to lysophosphatidylcholine acyl C20:3 |
| MUFA (PC) | Sum of mono-unsaturated glycerophosphocholins |
| MUFA (PC)/SFA (PC) | Ratio of MUFA (PC) to SFA (PC) |
| PUFA (PC) | Sum of poly-unsaturated glycerophosphocholins |
| PUFA (PC)/MUFA (PC) | Ratio of PUFA (PC) to MUFA (PC) |
| PUFA (PC)/SFA (PC) | PUFA (PC)/SFA (PC) |
| SFA (PC) | Sum of saturated glycerophosphocholins |
| Total (PC+SM) | Sum of choline-containing phospholipids |
| Total AC/C0 | Ratio of esterified to free carnitine |
| Total AC-DC/Total AC | Fraction of dicarboxyacylcarnitines of the total acylcarnitines |
| Total AC-OH/Total AC | Fraction of hydroxylated acylcarnitines of the total acylcarnitines |
| Total lysoPC | Sum of lysoglycerosphosphocholines |
| Total lysoPC/Total PC | Ratio of lysoglycerosphosphocholines to glycerosphosphocholines |
| Total PC | Sum of glycerosphosphocholines |
| Total PC aa | Sum of diacyl-glycerosphosphocholines |
| Total PC ae | Sum of glycerosphosphocholines plasmalogens |
| Total SM | Sum of ceramide phosphocholines (sphingomyelins) |
| Total SM/Total (SM+PC) | Fraction of ceramide phosphocholines (sphingomyelins) of total phospholipid pool |
| Total SM/Total PC | Ratio of ceramide phosphocholines (sphingomyelins) to total glycerophosphocholines |
| Total SM-non OH | Sum of non-hydroxylated ceramide phosphocholines (sphingomyelins) |
| Total SM-OH | Sum of hydroxylated ceramide phosphocholines (sphingomyelins) |
| Total SM-OH/Total SM-non OH | Ratio of hydroxylated to non-hydroxylated ceramide phosphocholines (sphingomyelins) |
