# Supplementary material for: Metabolomics in serum of patients with non-advanced age-related macular degeneration reveals aberrations in the glutamine pathway
Source: PLoS One. 2019 Jun 20;14(6):e0218457. doi: 10.1371/journal.pone.0218457 (PMC6586309; doi:10.1371/journal.pone.0218457)
Supplement: S3 Table — (DOCX) [file pone.0218457.s003.docx]

**S3 Table. Comparison of mean levels of all variables between cases and controls.**

| **Metabolite/Custom metabolic indicator** | **Cases** | | **Controls** | |  |
| --- | --- | --- | --- | --- | --- |
|  | **Mean (µM)** | **SD** | **Mean (µM)** | **SD** | **P-value*** |
| Ala | 455.94 | 87.82 | 459.40 | 107.19 | 0.79 |
| Arg | 111.46 | 26.45 | 102.97 | 23.05 | 0.09 |
| Asn | 51.78 | 8.43 | 50.35 | 10.58 | 0.18 |
| Asp | 20.27 | 5.40 | 21.12 | 6.61 | 0.57 |
| Cit | 39.18 | 9.68 | 36.56 | 10.35 | 0.12 |
| *Gln* | *746.33* | *99.19* | *695.01* | *108.33* | *7.67E-04* |
| Glu | 60.09 | 23.13 | 68.39 | 32.84 | 0.08 |
| *Gly* | *290.79* | *67.16* | *268.33* | *74.93* | *0.02* |
| His | 95.78 | 15.59 | 91.89 | 14.48 | 0.15 |
| Ile | 82.73 | 33.33 | 81.81 | 26.83 | 0.82 |
| Leu | 163.98 | 60.76 | 164.63 | 49.67 | 0.54 |
| Lys | 276.36 | 79.05 | 280.63 | 68.78 | 0.57 |
| Met | 24.73 | 7.60 | 23.31 | 6.12 | 0.35 |
| Orn | 107.93 | 28.19 | 108.97 | 28.49 | 0.67 |
| Phe | 73.17 | 14.15 | 75.94 | 14.59 | 0.25 |
| Pro | 263.57 | 79.02 | 254.44 | 73.30 | 0.57 |
| Ser | 133.70 | 23.06 | 129.78 | 24.78 | 0.34 |
| *Thr* | *123.59* | *24.81* | *115.68* | *24.15* | *0.04* |
| Trp | 62.97 | 12.93 | 62.46 | 13.18 | 0.86 |
| Tyr | 86.53 | 25.69 | 86.68 | 25.16 | 0.90 |
| Val | 284.13 | 79.55 | 290.03 | 64.60 | 0.30 |
| ADMA | 0.50 | 0.07 | 0.49 | 0.07 | 0.67 |
| Creatinine | 82.34 | 18.56 | 77.57 | 14.79 | 0.18 |
| Kynurenine | 3.04 | 0.79 | 2.87 | 0.66 | 0.35 |
| Putrescine | 0.14 | 0.06 | 0.13 | 0.06 | 0.26 |
| Sarcosine | 14.03 | 12.07 | 11.30 | 6.43 | 0.07 |
| Serotonin | 0.53 | 0.24 | 0.61 | 0.30 | 0.16 |
| Taurine | 137.31 | 36.98 | 140.50 | 45.98 | 0.73 |
| SDMA | 0.52 | 0.16 | 0.49 | 0.10 | 0.63 |
| AAA | 222.67 | 47.92 | 225.04 | 44.34 | 0.66 |
| ADMA/Arg | 4.68E-03 | 1.15E-03 | 4.99E-03 | 1.44E-03 | 0.26 |
| Arg/(Arg+Orn) | 0.51 | 0.08 | 0.49 | 0.09 | 0.07 |
| BCAA | 530.89 | 170.20 | 536.47 | 137.31 | 0.44 |
| Cit/Arg | 0.37 | 0.11 | 0.37 | 0.12 | 0.99 |
| *Cit/Orn* | *0.38* | *0.11* | *0.35* | *0.12* | *0.04* |
| Essential AA | 1091.63 | 279.79 | 1094.47 | 223.40 | 0.53 |
| Fisher ratio | 2.36 | 0.37 | 2.38 | 0.31 | 0.46 |
| *Glu/Gln* | *0.08* | *0.03* | *0.10* | *0.06* | *0.02* |
| Glucogenic AA | 880.57 | 116.55 | 857.46 | 159.62 | 0.14 |
| *Glutaminolysis* | *0.72* | *0.13* | *0.80* | *0.19* | *0.01* |
| Gly/Arg | 2.76 | 0.97 | 2.73 | 0.98 | 0.87 |
| *Gly/Gln* | *0.39* | *0.10* | *0.39* | *0.11* | *0.70* |
| Gly/His | 3.11 | 0.86 | 2.96 | 0.84 | 0.16 |
| Gly/Ser | 2.21 | 0.54 | 2.08 | 0.44 | 0.19 |
| Glycolysis | 880.57 | 116.55 | 857.46 | 159.62 | 0.14 |
| Kynurenine/Trp | 0.05 | 0.02 | 0.05 | 0.01 | 0.49 |
| Non essential AA | 2463.54 | 290.46 | 2373.85 | 324.36 | 0.05 |
| Orn/Arg | 1.02 | 0.35 | 1.12 | 0.40 | 0.07 |
| Orn/Ser | 0.82 | 0.21 | 0.85 | 0.21 | 0.22 |
| Putrescine/Orn | 1.43E-03 | 7.47E-04 | 1.31E-03 | 7.44E-04 | 0.34 |
| SDMA/Arg | 4.90E-03 | 1.89E-03 | 4.99E-03 | 1.80E-03 | 0.73 |
| Serotonin/Trp | 0.01 | 4.07E-03 | 0.01 | 0.01 | 0.25 |
| Thr/Ser | 0.94 | 0.19 | 0.91 | 0.18 | 0.22 |
| Total AA | 3555.29 | 485.58 | 3468.36 | 489.74 | 0.30 |
| Total DMA/Arg | 0.01 | 2.87E-03 | 0.01 | 3.05E-03 | 0.31 |
| Tyr/Phe | 1.18 | 0.21 | 1.15 | 0.27 | 0.28 |
| C0 | 41.90 | 7.63 | 40.84 | 7.87 | 0.32 |
| C10 | 0.29 | 0.15 | 0.25 | 0.09 | 0.05 |
| C10:1 | 0.15 | 0.05 | 0.14 | 0.05 | 0.13 |
| C12 | 0.11 | 0.04 | 0.10 | 0.03 | 0.06 |
| C12:1 | 0.12 | 0.03 | 0.12 | 0.04 | 0.46 |
| C14 | 0.05 | 0.01 | 0.05 | 0.01 | 0.59 |
| C14:1 | 0.07 | 0.03 | 0.07 | 0.03 | 0.24 |
| C14:2 | 0.03 | 0.02 | 0.03 | 0.01 | 0.65 |
| C16 | 0.13 | 0.03 | 0.13 | 0.03 | 0.73 |
| C18 | 0.06 | 0.01 | 0.06 | 0.01 | 0.74 |
| C18:1 | 0.15 | 0.03 | 0.15 | 0.04 | 0.89 |
| C18:2 | 0.05 | 0.02 | 0.05 | 0.02 | 0.95 |
| C2 | 6.38 | 2.10 | 6.48 | 2.70 | 0.70 |
| C3 | 0.41 | 0.15 | 0.42 | 0.15 | 0.56 |
| C4-OH (C3-DC) | 0.05 | 0.02 | 0.06 | 0.03 | 0.27 |
| C4 | 0.23 | 0.09 | 0.22 | 0.07 | 0.86 |
| C4:1 | 0.01 | 2.93E-03 | 0.01 | 2.85E-03 | 0.13 |
| C6 (C4:1-DC) | 0.07 | 0.03 | 0.06 | 0.02 | 0.50 |
| C5 | 0.14 | 0.05 | 0.15 | 0.04 | 0.38 |
| *C5:1* | *0.04* | *0.01* | *0.04* | *0.01* | *0.03* |
| C5:1-DC | 0.02 | 3.98E-03 | 0.02 | 4.94E-03 | 0.35 |
| C5-DC (C6-OH) | 0.02 | 0.01 | 0.02 | 4.61E-03 | 0.68 |
| C8 | 0.16 | 0.08 | 0.14 | 0.04 | 0.10 |
| C9 | 0.08 | 0.04 | 0.07 | 0.03 | 0.32 |
| lysoPC a C16:0 | 82.08 | 17.83 | 80.99 | 15.09 | 0.82 |
| lysoPC a C16:1 | 2.63 | 0.72 | 2.66 | 0.69 | 0.71 |
| lysoPC a C17:0 | 1.61 | 0.40 | 1.58 | 0.39 | 0.59 |
| lysoPC a C18:0 | 23.57 | 5.70 | 23.22 | 5.48 | 0.71 |
| lysoPC a C18:1 | 17.86 | 4.52 | 17.02 | 4.38 | 0.25 |
| lysoPC a C18:2 | 33.07 | 12.15 | 30.11 | 11.33 | 0.13 |
| lysoPC a C20:3 | 1.91 | 0.73 | 1.91 | 0.59 | 0.66 |
| lysoPC a C20:4 | 5.16 | 1.86 | 5.43 | 1.82 | 0.31 |
| *lysoPC a C24:0* | *0.14* | *0.04* | *0.13* | *0.04* | *0.02* |
| lysoPC a C26:0 | 0.18 | 0.07 | 0.17 | 0.06 | 0.36 |
| lysoPC a C26:1 | 0.09 | 0.03 | 0.08 | 0.03 | 0.09 |
| lysoPC a C28:0 | 0.28 | 0.16 | 0.24 | 0.11 | 0.17 |
| lysoPC a C28:1 | 0.35 | 0.10 | 0.32 | 0.11 | 0.11 |
| PC aa C24:0 | 0.12 | 0.07 | 0.10 | 0.05 | 0.08 |
| *PC aa C28:1* | *3.35* | *0.76* | *3.04* | *0.73* | *0.01* |
| PC aa C30:0 | 5.13 | 1.64 | 5.04 | 1.74 | 0.79 |
| PC aa C32:0 | 18.92 | 3.68 | 18.20 | 3.69 | 0.17 |
| PC aa C32:1 | 23.11 | 8.82 | 24.49 | 10.60 | 0.58 |
| PC aa C32:2 | 5.93 | 2.08 | 5.86 | 2.36 | 0.67 |
| PC aa C32:3 | 0.64 | 0.17 | 0.61 | 0.18 | 0.33 |
| PC aa C34:1 | 280.85 | 60.37 | 279.64 | 63.96 | 0.74 |
| PC aa C34:2 | 671.69 | 146.92 | 641.22 | 147.45 | 0.18 |
| PC aa C34:3 | 24.39 | 6.58 | 23.60 | 7.09 | 0.41 |
| PC aa C34:4 | 2.55 | 0.87 | 2.58 | 0.85 | 0.80 |
| *PC aa C36:0* | *3.97* | *1.04* | *3.59* | *1.00* | *0.04* |
| PC aa C36:1 | 47.82 | 11.56 | 47.51 | 11.90 | 0.78 |
| PC aa C36:2 | 338.97 | 71.27 | 323.18 | 72.29 | 0.09 |
| PC aa C36:3 | 168.88 | 35.18 | 163.33 | 34.25 | 0.27 |
| PC aa C36:4 | 244.67 | 59.97 | 253.83 | 57.01 | 0.42 |
| PC aa C36:5 | 42.86 | 21.81 | 39.52 | 16.65 | 0.65 |
| PC aa C36:6 | 1.47 | 0.57 | 1.41 | 0.58 | 0.56 |
| *PC aa C38:0* | *3.62* | *0.92* | *3.26* | *0.90* | *0.02* |
| PC aa C38:1 | 0.91 | 0.58 | 0.79 | 0.58 | 0.14 |
| PC aa C38:3 | 51.87 | 13.31 | 52.21 | 13.54 | 0.93 |
| PC aa C38:4 | 118.93 | 31.31 | 125.37 | 32.81 | 0.34 |
| PC aa C38:5 | 67.39 | 17.06 | 66.52 | 14.07 | 0.97 |
| PC aa C38:6 | 107.66 | 34.39 | 102.09 | 28.18 | 0.55 |
| *PC aa C40:2* | *0.36* | *0.11* | *0.33* | *0.09* | *0.04* |
| *PC aa C40:3* | *0.60* | *0.14* | *0.56* | *0.13* | *0.03* |
| PC aa C40:4 | 3.26 | 0.89 | 3.28 | 0.80 | 0.88 |
| PC aa C40:5 | 9.59 | 2.64 | 9.70 | 2.32 | 0.57 |
| PC aa C40:6 | 33.63 | 9.99 | 32.92 | 9.82 | 0.62 |
| PC aa C42:0 | 0.63 | 0.16 | 0.58 | 0.14 | 0.10 |
| PC aa C42:1 | 0.33 | 0.08 | 0.30 | 0.07 | 0.12 |
| PC aa C42:2 | 0.23 | 0.06 | 0.21 | 0.05 | 0.07 |
| PC aa C42:4 | 0.17 | 0.03 | 0.16 | 0.03 | 0.05 |
| PC aa C42:5 | 0.36 | 0.09 | 0.34 | 0.08 | 0.35 |
| PC aa C42:6 | 0.53 | 0.13 | 0.49 | 0.10 | 0.08 |
| PC ae C30:0 | 0.47 | 0.12 | 0.45 | 0.13 | 0.30 |
| PC ae C30:1 | 0.14 | 0.11 | 0.12 | 0.09 | 0.40 |
| PC ae C30:2 | 0.11 | 0.03 | 0.11 | 0.03 | 0.21 |
| PC ae C32:1 | 3.57 | 0.70 | 3.37 | 0.76 | 0.08 |
| PC ae C32:2 | 0.92 | 0.21 | 0.87 | 0.23 | 0.12 |
| PC ae C34:0 | 1.94 | 0.48 | 1.90 | 0.51 | 0.51 |
| PC ae C34:1 | 12.16 | 2.74 | 11.57 | 2.55 | 0.19 |
| PC ae C34:2 | 15.66 | 3.79 | 14.66 | 3.83 | 0.13 |
| PC ae C34:3 | 10.99 | 2.91 | 10.15 | 3.32 | 0.06 |
| PC ae C36:0 | 1.46 | 0.45 | 1.42 | 0.46 | 0.50 |
| PC ae C36:1 | 9.68 | 2.22 | 9.44 | 2.12 | 0.53 |
| PC ae C36:2 | 18.36 | 4.19 | 17.63 | 4.18 | 0.27 |
| PC ae C36:3 | 9.82 | 2.28 | 9.24 | 2.41 | 0.11 |
| PC ae C36:4 | 18.69 | 4.75 | 18.33 | 4.89 | 0.38 |
| PC ae C36:5 | 14.13 | 3.16 | 13.87 | 3.91 | 0.45 |
| PC ae C38:0 | 3.15 | 1.05 | 2.87 | 0.90 | 0.11 |
| PC ae C38:1 | 0.74 | 0.49 | 0.68 | 0.46 | 0.48 |
| PC ae C38:2 | 2.15 | 0.56 | 2.08 | 0.56 | 0.31 |
| PC ae C38:3 | 4.65 | 1.13 | 4.53 | 1.05 | 0.41 |
| PC ae C38:4 | 14.24 | 3.37 | 14.37 | 3.07 | 0.75 |
| PC ae C38:5 | 18.14 | 3.81 | 17.60 | 3.93 | 0.20 |
| PC ae C38:6 | 8.86 | 2.04 | 8.38 | 2.34 | 0.15 |
| *PC ae C40:1* | *1.60* | *0.42* | *1.46* | *0.31* | *0.04* |
| PC ae C40:2 | 1.94 | 0.43 | 1.78 | 0.42 | 0.05 |
| PC ae C40:3 | 1.07 | 0.25 | 1.01 | 0.22 | 0.10 |
| PC ae C40:4 | 2.28 | 0.50 | 2.26 | 0.45 | 0.62 |
| PC ae C40:5 | 3.46 | 0.72 | 3.40 | 0.62 | 0.47 |
| PC ae C40:6 | 5.21 | 1.20 | 4.94 | 1.12 | 0.19 |
| PC ae C42:1 | 0.39 | 0.07 | 0.37 | 0.07 | 0.07 |
| PC ae C42:2 | 0.62 | 0.15 | 0.57 | 0.14 | 0.08 |
| *PC ae C42:3* | *0.77* | *0.18* | *0.70* | *0.15* | *0.04* |
| PC ae C42:4 | 0.77 | 0.19 | 0.72 | 0.16 | 0.15 |
| PC ae C42:5 | 1.81 | 0.36 | 1.78 | 0.35 | 0.41 |
| PC ae C44:3 | 0.12 | 0.03 | 0.11 | 0.03 | 0.26 |
| PC ae C44:4 | 0.33 | 0.08 | 0.31 | 0.07 | 0.13 |
| PC ae C44:5 | 1.33 | 0.35 | 1.31 | 0.30 | 0.61 |
| PC ae C44:6 | 1.09 | 0.25 | 1.06 | 0.27 | 0.30 |
| SM (OH) C14:1 | 6.88 | 1.63 | 6.38 | 1.50 | 0.13 |
| SM (OH) C16:1 | 3.43 | 0.78 | 3.28 | 0.70 | 0.27 |
| SM (OH) C22:1 | 10.65 | 2.31 | 10.26 | 2.43 | 0.33 |
| SM (OH) C22:2 | 9.91 | 2.31 | 9.45 | 2.30 | 0.29 |
| SM (OH) C24:1 | 1.03 | 0.23 | 1.00 | 0.26 | 0.74 |
| SM C16:0 | 112.00 | 18.45 | 105.11 | 19.58 | 0.06 |
| SM C16:1 | 16.44 | 3.25 | 15.61 | 3.25 | 0.07 |
| SM C18:0 | 20.98 | 4.85 | 19.94 | 4.51 | 0.20 |
| SM C18:1 | 10.64 | 2.59 | 10.32 | 2.62 | 0.28 |
| SM C20:2 | 0.50 | 0.14 | 0.46 | 0.13 | 0.05 |
| SM C24:0 | 16.08 | 3.09 | 15.37 | 3.77 | 0.22 |
| SM C24:1 | 36.78 | 6.79 | 34.82 | 7.33 | 0.15 |
| SM C26:0 | 0.22 | 0.12 | 0.22 | 0.13 | 0.58 |
| *SM C26:1* | *0.31* | *0.08* | *0.28* | *0.09* | *0.02* |
| H1 | 5215.13 | 1491.50 | 5100.92 | 1569.56 | 0.65 |
| (C2+C3)/C0 | 0.17 | 0.06 | 0.17 | 0.07 | 0.89 |
| C18/C18:1 | 0.42 | 0.08 | 0.43 | 0.09 | 0.92 |
| C2/C0 | 0.16 | 0.06 | 0.16 | 0.07 | 0.99 |
| C3/C4 | 1.88 | 0.51 | 1.95 | 0.48 | 0.47 |
| C4/C0 | 0.01 | 2.31E-03 | 0.01 | 1.60E-03 | 0.47 |
| C4/C5 | 1.65 | 0.64 | 1.53 | 0.42 | 0.23 |
| CPT-I ratio | 4.81E-03 | 1.25E-03 | 4.85E-03 | 1.36E-03 | 0.96 |
| lysoPC a C16:0/lysoPC a C16:1 | 32.18 | 5.96 | 31.49 | 6.36 | 0.49 |
| lysoPC a C20:4/lysoPC a C20:3 | 2.82 | 0.72 | 2.93 | 0.85 | 0.37 |
| MUFA (PC) | 384.21 | 83.00 | 382.40 | 88.20 | 0.71 |
| MUFA (PC)/SFA (PC) | 9.45 | 1.39 | 9.92 | 1.72 | 0.21 |
| PUFA (PC) | 2054.08 | 334.53 | 2001.17 | 346.14 | 0.23 |
| PUFA (PC)/MUFA (PC) | 5.46 | 0.84 | 5.38 | 1.01 | 0.64 |
| PUFA (PC)/SFA (PC) | 50.91 | 6.60 | 52.41 | 8.41 | 0.39 |
| SFA (PC) | 40.89 | 7.88 | 38.82 | 7.47 | 0.08 |
| Total (PC+SM) | 2724.97 | 430.31 | 2654.83 | 431.69 | 0.23 |
| Total AC/C0 | 0.23 | 0.08 | 0.23 | 0.08 | 0.76 |
| Total AC-DC/Total AC | 0.03 | 0.01 | 0.03 | 0.01 | 0.08 |
| Total AC-OH/Total AC | 0.02 | 4.16E-03 | 0.02 | 0.01 | 0.39 |
| Total lysoPC | 172.10 | 38.13 | 167.06 | 34.32 | 0.44 |
| Total lysoPC/Total PC | 0.07 | 0.02 | 0.07 | 0.01 | 0.90 |
| Total PC | 2479.11 | 403.90 | 2422.40 | 406.67 | 0.29 |
| Total PC aa | 2285.51 | 377.89 | 2236.17 | 382.11 | 0.32 |
| Total PC ae | 193.64 | 33.98 | 186.22 | 34.11 | 0.19 |
| Total SM | 245.85 | 40.54 | 232.53 | 43.07 | 0.08 |
| Total SM/Total (SM+PC) | 0.09 | 0.01 | 0.09 | 0.01 | 0.38 |
| Total SM/Total PC | 0.10 | 0.01 | 0.10 | 0.02 | 0.40 |
| Total SM-non OH | 213.97 | 34.51 | 202.10 | 37.28 | 0.06 |
| Total SM-OH | 31.91 | 6.73 | 30.37 | 6.64 | 0.25 |
| Total SM-OH/Total SM-non OH | 0.15 | 0.02 | 0.15 | 0.02 | 0.81 |

*Using wilcoxon signed-rank tests. Variables in italic have a P-value < 0.05.
